# MYmind: a Concurrent Group-Based Mindfulness Intervention for Youth with Autism and Their Parents

**DOI:** 10.1007/s12671-019-01107-9

**Published:** 2019-02-22

**Authors:** Sandra Salem-Guirgis, Carly Albaum, Paula Tablon, Priscilla Burnham Riosa, David B. Nicholas, Irene E. Drmic, Jonathan A. Weiss

**Affiliations:** 1 School of Health & Wellness, George Brown College, Toronto, Canada; 2 Department of Psychology, York University, Toronto, Canada; 3 Department of Applied Disability Studies, Brock University, St. Catherines, Canada; 4 Faculty of Social Work, Central and Northern Alberta Region, University of Calgary, Edmonton, Canada; 5 Ron Joyce Children’s Health Centre, Hamilton Health Sciences, Hamilton, Ontario Canada

**Keywords:** MYmind, Autism, Parents, Youth, Mindfulness

## Abstract

**Objectives:**

The current study evaluated the use of MYmind, a concurrent mindfulness program in which youth with autism and their parents simultaneously receive group specific mindfulness training. Youth with autism can experience emotional and behavioral challenges, which are associated with parental stress. Mindfulness-based programs are emerging as a promising support for these challenges, for both children and parents. While two studies have documented the use of concurrent parent-child programs, neither involve control conditions.

**Methods:**

Using a within-subject repeated measures design with a baseline component, 23 parent-child dyads were assessed on mindfulness, mental health, and youth emotion regulation and autism symptoms. Participants also rated their perceived improvement on a social validity questionnaire.

**Results:**

There was improvement in youth autism symptoms, emotion regulation, and adaptive skills, and in parent reports of their own mindfulness following the program. There was also some indication of a waitlist effect for parent mental health, but not for other outcome variables. Participant feedback was mainly positive.

**Conclusions:**

MYmind has the potential to contribute to emotion regulation and adaptability in youth with autism, and mindfulness in parents, though more rigorous controlled trials are needed.

Individuals with autism exhibit stereotyped interests and behaviors, and significant impairments in various domains of social functioning (American Psychiatric Association 2013). Recent prevalence studies estimate that between 1 in 80 and 1 in 45 children may have autism (Zablotsky et al. 2015). Young people with autism frequently experience emotional and behavioral challenges (Hofvander et al. 2009; Simonoff et al. 2008), such as aggression, emotional outbursts, anxiety, and depression (Leyfer et al. 2006; Simonoff et al. 2008; Totsika et al. 2011). Research suggests that difficulties with emotion regulation may play a part in these frequent comorbidities (Khor et al. 2014; Mazefsky et al. 2014; Samson et al. 2015). These challenges can be a poignant source of stress for parents, and there is evidence supporting a bi-directional relationship between parent and child functioning (Zaidman-Zait et al. 2014). Overall, parents of children with autism experience higher levels of stress as well as a higher incidence of depression and anxiety compared to other families (Hayes and Watson 2013).

There is a growing body of research suggesting that mindfulness-based training (MBT) for youth with autism and their parents may improve youth and parent psychosocial functioning. MBT include various programs (e.g., mindfulness-based stress reduction and mindfulness-based cognitive therapy) and meditation techniques that aim to increase *mindfulness*—a present-oriented awareness, accompanied by non-judgmental observation, and non-reactive responding (Kabat-Zinn 2003). There is evidence that MBT is effective in reducing psychopathology in youth and adults without autism (Baer 2003; Burke 2010; Hofmann et al. 2010; Segal et al. 2013), likely through increased insight and acceptance of personal experiences, reduced ruminative thought patterns, and strengthened cognitive and behavioral flexibility (Gu et al. 2015).

Recent systematic reviews describe the current state of the evidence with regard to MBT for people with autism and their parents. Cachia et al. (2015) examined 10 MBT programs delivered to parents of children with autism: six within-case pre-post-follow-up designs, three between-group designs, and one pre-post design. The authors found evidence for improved parental stress and well-being, as well as indirect effects of parent mindfulness training on child behavior (e.g., externalizing behavior problems, child compliance, and aggression), theory of mind, central coherence, and executive functioning. At the same time, the overall quality of the literature was deemed to be low, indicating a need for additional research utilizing control conditions. Cachia et al. (2016) examined six MBT programs delivered directly to individuals with autism, across the lifespan: three within-group pre-post-follow-up designs, two within-case multiple baseline designs, and one randomized wait list controlled trial. The results from these studies suggest that MBT may positively impact internalizing and externalizing problems, autism symptomatology, and psychological well-being of individuals with autism. Additionally, results from these and other studies suggest that MBT may positively impact emotion regulation difficulties often experienced by people with autism such as inflexibility, rumination, reappraisal, and perspective taking. MBT programs directly target these factors through training in skills such as directed attention and acceptance of thoughts and emotions (Conner and White 2018; Eack et al. 2013; Rieffe et al. 2011; Samson et al. 2014).

Within the array of MBT programs for people with autism, one novel approach, MYmind, provides group-based mindfulness training for parents and mindfulness training for their children in a concurrent manner, with the aim of supporting the child, the parent, and the parent-child relationship (de Bruin et al. 2015; Ridderinkhof et al. 2018). Two studies stemming from the same research group have evaluated MYmind for youth with autism and their parents. de Bruin et al. (2015) employed a pre-post-follow-up design to assess the effects of MYmind on 23 youth (11–23 years old) with autism without intellectual disability and 29 of their parents (40–59 years old). Participants were assessed 1 week prior to the start of the program, 1 week post-program, and 9 weeks post-program. Results indicated positive change in parent reports of youth autism symptoms (i.e., social responsiveness, communication, cognition, and motivation), mindful parenting (e.g., decreased reactivity and increased ability to observe and describe), parenting style, and quality of life. Youth reported increased quality of life and decreased rumination, though no changes were reported for worrying, autism symptoms, and mindfulness. Youth specifically noted that they found the breathing meditation and walking meditation as most useful.

A more recent study of MYmind used a pre-post-follow-up (2-month and 1-year follow-ups), with 8–19 year olds with autism without intellectual disability (Ridderinkhof et al. 2018). Results from this study tended to replicate those reported in de Bruin et al. (2015), including a decrease in parent-reported youth autism symptoms (specifically social communication problems) and improvements in internalizing and externalizing symptoms. Youth reported decreased rumination and a small but nonsignificant increase in overall well-being. Additionally, parents reported improvements in their own social communication, emotional and behavioral functioning, parenting-related stress, mindful parenting, and self-compassion. Changes were mostly maintained at 2-month and 1-year follow-ups, with some additional improvements seen in youth outcomes. A qualitative analysis for both children and parents resulted in three main emerging themes: *improved mindfulness skills*, *improved well-being*, and, to a much smaller extent, *little to no change*. Though results are promising, neither of these studies included a comparison condition to rule out changes due to the passage of time or re-assessment alone, either in terms of a within-subject waitlist or experimental control group. MYmind has also been applied to youth with attention-deficit/hyperactivity disorder and their parents (the original target population), with similar improvements in youth and parents (youth reported improvements in attention, hyperactivity/impulsivity, anxiety, social behavior, problem behavior, awareness and executive functioning, and parent improvements in parenting stress, over-reactivity, impulsivity, awareness, and attention; Van de Weijer-Bergsma et al. 2011; Van der Oord et al. 2012).

Building on the past MYmind autism literature, the current study reports on the outcomes of MYmind for adolescents and young adults (subsequently referred to as “youth”) with autism and their parents, by adding a baseline period, along with the traditional program and follow-up periods. We also sought to add to the literature by attempting to replicate prior results as an independent research team. We hypothesized that the program period would result in improvements in youth and parent mental health symptoms and mindfulness, youth autism symptoms and emotion regulation, and parent stress, which would also appear when comparing pre-treatment scores with follow-up. We expected no change during the baseline period.

## Methods

### Participants

The final sample consisted of 23 parent-youth dyads, who participated in a 10-session MYmind program in the Greater Toronto Area between 2014 and 2017. Youth were between the ages of 12 and 23 years (82.6% male; *M*_age_ = 15.65, *SD* = 2.57) and were living with their parents. This age range was a direct reflection of that described in de Bruin et al. (2015). Parents were between 40 and 59 years of age (87% female; *M*_age_ = 50.05, *SD* = 5.25). On average, youth attended 90.8% of sessions (*SD* = 11.76, range 60–100%), and parents attended 91.3% (*SD* = 9.00, range 70–100%). Additional participant characteristics are presented in Table 1.

**Table 1:** Adolescent and parent characteristics

	Youth (*n* = 23)	Parents (*n* = 23)
Gender (% male)	19 (82.6)	3 (13.0)
Age (*M*, *SD*)	15.65 (2.57)	50.05 (5.25)
Range	12–23	40–59
FSIQ-2 composite (*M*, *SD*)	103.96 (12.98)	–
Range	74–123	–
SRS-2 *t*-score (*M*, *SD*)	93.27 (24.12)	–
Range	48–136	–
SCQ (*M*, *SD*)	17.96 (6.51)	–
Range	14–27	–
Ethnicity (% White/Caucasian)	71.4	71.4
Psychotropic medication use (% yes)	12 (52.2)	–
Disclosed education (%)		
In grade 7–8	4 (17.4)	–
In grade 9–12	17 (73.9)	–
High school graduate	1 (4.3)	1 (4.3)
Some college	1 (4.3)	2 (8.7)
College diploma	–	2 (8.7)
University degree	–	9 (39.1)
Marital status (% married/common law)	–	18 (78.3)
Disclosed employment status (%)		
Full time	–	6 (42.9)
Part time	–	3 (21.4)
Disabled (permanently or temporarily)/sick leave	–	2 (14.3)
Self-employed	–	3 (21.4)

*FSIQ-2*, Wechlser Abbreviated Scale of Intelligence Full Scale IQ – 2 subscales; *SRS-2*, Social Responsiveness Scale, 2nd Edition; *SCQ*, Social Communication Questionnaire-Lifetime Version

Families were eligible to participate in the group if the youth met the following inclusion criteria: (a) confirmation of autism diagnosis from a report provided by a licensed clinician, (b) parents reported youth symptoms that were above clinical cutoff score of 14 on the Social Communication Questionnaire – Lifetime Version (SCQ; Rutter et al. 2003) or a *T*-score greater than 60 on the Social Responsiveness Scale, Second Edition (SRS-2; Constantino and Gruber 2012); (c) youth and parent willingness and interest to attend MYmind sessions and research appointments. Exclusion criteria were (a) youth with a diagnosis of an intellectual disability or significantly below average intellectual functioning on the Wechsler Abbreviated Scale of Intelligence-Second Edition (IQ < 70, WASI-II; Wechsler 2011); (b) parent or youth current involvement in overlapping psychotherapy; (c) aggressive/self-injurious behaviors that would be a cause for concern in a group setting; or (d) the same parent was not able to consistently attend parent sessions.

**Fig. 1:**
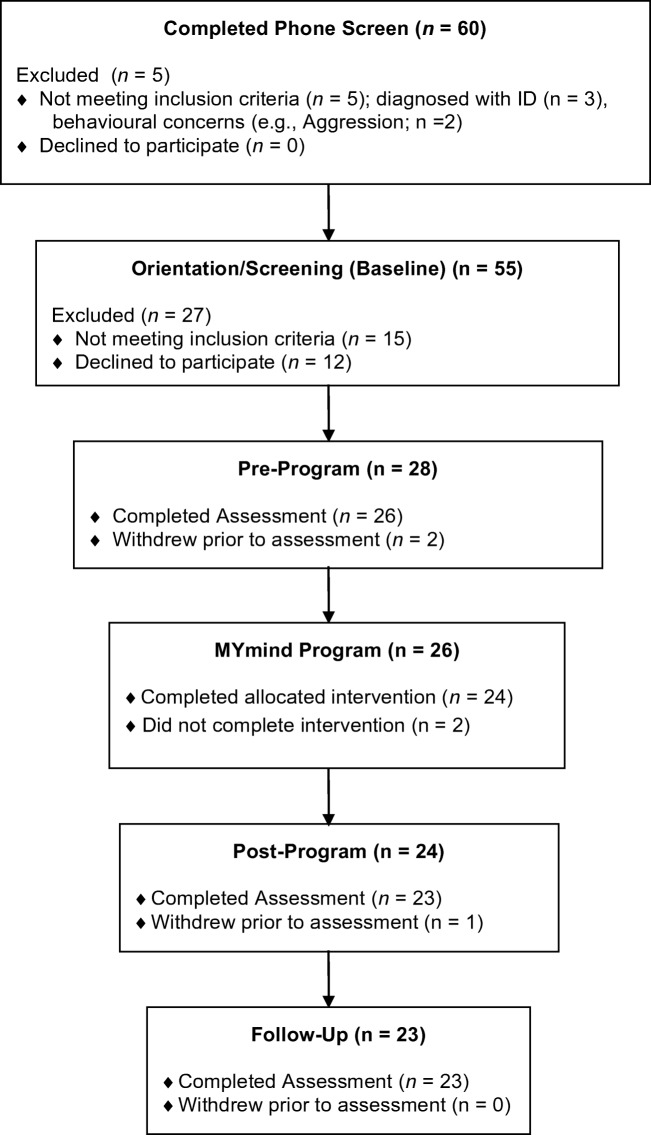
MYmind participant recruitment

Participants were recruited over the course of 3 years, through local autism service e-newsletters and website postings. Initial phone screens were completed with 60 parents, 55 of whom were invited to an orientation appointment. During this appointment, youth and parents each provided informed consent/assent, youth were screened for IQ using the WASI-II (Wechsler 2011), and parents were asked to complete the SRS-2 (Constantino and Gruber 2012), SCQ (Rutter et al. 2003), and a demographic questionnaire. Each parent-youth dyad was individually interviewed by lead facilitators of the respective parent/youth groups to assess motivation to engage in the program, and willingness and interest to participate. At this time, parents were provided with the MYmind program schedule, and the expectations of the program were discussed. Following orientation, 12 families declined to participate in the study, and 15 did not meet inclusion criterion. The remaining 28 families were invited to participate in the program, of which two withdrew prior to the start of the program, two did not complete the program, and one completed it but withdrew from the study prior to the post-program assessment (see Fig. 1).

### Procedures

#### Facilitators

All lead MYmind facilitators obtained instructor certification by completing a 5-day MYmind training workshop by the program developers (Drs. Susan Bögels and Esther de Bruin), a separate standard Mindfulness Based Cognitive Therapy or Mindfulness Based Stress Reduction course, and a 3-day-long silent mindfulness retreat. Two clinical psychologists and three behavioral consultants participated as lead facilitators across 3 years (*M* years of experience working with autism population = 14.0, *SD* = 9.31), who were selected based on competency and availability and expressed interest in facilitating MYmind groups. Further, two psychology post-doctoral fellows and six clinical psychology graduate students co-facilitated the MYmind sessions (*M* years of experience with population = 4.31, *SD* = 3.90).

#### Program

The current MYmind program was developed specifically for children and adolescents with autism and their parents. The program aims to improve awareness, distress tolerance, and self-control in youth with autism through various mindfulness techniques (e.g., meditations, breathing techniques, yoga poses). The concurrent parent group focuses on the impact of reactivity, attending to youth non-judgmentally, and accepting their children and their own feelings about parenting (de Bruin et al. 2015). Youth and parents met as separate groups for nine weekly sessions and one booster session, which occurred 9 weeks post-program (1.5 h each). Each group was led by a trained facilitator and was further supported by a co-facilitator.

Sessions involved elements of mindfulness and cognitive behavior therapy. Each week, a core set of mindfulness-based skills were practiced, such as mindful eating, body scanning, guided breathing, and meditation exercises. Parents were taught mindful parenting skills and learned how to support and guide their children through mindfulness practice. The general sequence of each session for parents and youth flowed accordingly: breathing meditation, review of previous week’s home practice, introduction of session’s theme, short semi-structured break (for youth only), a second type of meditation and/or yoga, review of the following week’s home practice, followed by a final breathing meditation. Weekly guided meditation recordings were sent to families for home practice to support generalizability of the skills learned in session.

#### Procedural Integrity

Sessions were video-recorded for the purpose of examining procedural integrity after completion of the trial. A checklist of content items for each session was developed based on information from the MYmind manual. It included specific activities that were listed on the first page of each session in the manual (e.g., breathing space and inquiry, walking meditation) and non-specific session items described in facilitator training (e.g., setup, materials, and facilitator’s behavior). If an item referred to another group (i.e., if list included a combined parent and child activity), they were included in both checklists. This resulted in unique checklists with a range of items (9–15 items) depending on session requirements. Raters scored items on the checklist as either complete (if the item was completed as described in the manual) or incomplete (if the item was either partially completed or not completed within the session). A total of 30% of the recorded sessions (*n* = 30) were evaluated for procedural integrity, 46.7% (*n* = 14) of which were double scored to check for inter-rater reliability. Sessions were chosen for review randomly and included an evaluation of both youth and parent sessions. Raters achieved a mean of 97.3% (range 89–100%) inter-rater reliability across double scored sessions using point-by-point correspondence calculations. Across 30 sessions, facilitators/co-facilitators achieved an average of 80.5% procedural integrity, with a median score of 85% (range 45–100%). Scores were negatively skewed as a result of 7 sessions that were below 75% adherence. Common issues included items not being completed and meditations and activities being replaced by something other than what was noted in the manual (though they may have targeted the same thing), and all elements of the item not being completed as described (e.g., missing inquiry after a meditation, missing activity within introduction of topic). Due to the small number of items on each checklist, overall percentages were greatly affected by each incomplete item. In addition to this, for one of the sessions reviewed, the facilitator completed the session out of order.

#### Design

A within-subject repeated measures design across three cohorts was used to evaluate program outcomes. Data were collected from youth and their parents at four separate time points: (1) 10 weeks prior to starting the program (baseline); (2) 1 week prior to starting the program (pre-program); (3) 1 week following the final session (post-program); and (4) 10 weeks following the final session (follow-up).

The York University Research Ethics Board provided ethical approval for this study. All procedures were in accordance with the ethical standards of the York University Research Ethics Board. Informed consent was obtained from all participants and informed assent was obtained from the youth if they were under 16 years of age.

### Measures

#### Youth Mental Health

The *Behavior Assessment System for Children, Second Edition* (*BASC-2*; Reynolds and Kamphaus 2004) Parent Rating Scale (*BASC-2 PRS*) and Self-Report of Personality (*BASC-2 SRP*) were used to assess parent report and self-report of youth psychopathology. Items on this 160-item parent report questionnaire describe specific youth behaviors that are rated on frequency of occurrence using a 4-point response scale (1 = “Never” to 4 = “Almost Always”), producing four composite scores: Adaptive Skills, Behavioral Symptoms Index, Externalizing Problems, and Internalizing Problems. Although not developed for use specifically with autism populations, the BASC-2 PRS has been previously used to assess emotional and behavioral problems in research involving youth with autism (Mahan and Matson 2011; Volker et al. 2010). The BASC-2 SRP is a 179-item self-report questionnaire for adolescents 12–21 years. It is a combination of true/false questions and 4-point response scale (1 = “Never” to 4 = “Almost Always”), yielding five composite scores: School Problems, Internalizing Problems, Inattention/Hyperactivity, Emotional Symptoms Index, and Personal Adjustment. The BASC-2 SRP has been used to study psychosocial functioning in adolescents with autism through self-report (Foley-Nicpon et al. 2010)*.*

#### Youth Emotion Regulation

The Ruminative Response Scale (RRS; Reynor et al. 2003) was used to examine rumination on two subscales: Reflection and Brooding. The RRS consists of 22 items rated on a 4-point response scale (1 = “Almost never” to 4 = “Almost always”), indicating the frequency at which the youth engages in each behavior. For the current study, the internal consistencies were good for both subscales (Reflection, Cronbach’s *α* = .81; Brooding, Cronbach’s *α* = .90).

The Emotion Regulation Checklist (ERC; Shields and Cicchetti 1997) was used to assess parent perceptions of youth emotionality and regulation. Items were rated by parents on a 4-point response scale (1 = “Rarely/never” to 4 = “Almost always”), yielding two subscale scores: Emotion Regulation and Lability/Negativity. Internal consistency was acceptable for Lability/Negativity (Cronbach’s *α* = .75), and good for Emotion Regulation (Cronbach’s *α* = .85).

The Emotion Regulation Questionnaire-Child (ERQ-CA; Gullone and Taffe 2012) was used to assess youth’s self-report of two emotion regulation strategies: Cognitive Reappraisal and Expressive Suppression. The ERQ-CA consists of 10 questions rated using a 5-point scale (1 = “Strongly disagree” to 5 = “Strongly agree”). Internal consistencies were good for both Cognitive Reappraisal (Cronbach’s *α* = .85) and Expressive Suppression (Cronbach’s *α* = .84).

#### Youth Mindfulness

The Child and Adolescent Mindfulness Measure (CAMM; Greco et al. 2011) is a 10-item questionnaire used to assess present awareness and mindfulness of adolescents. Each item was rated using a 5-point scale (0 = “Never true” to 5 = “Always true”), and a total score was computed by averaging responses across the ten items. The CAMM has been reported to have good construct and incremental validity (Cronbach’s *α* = .81). In their recent evaluation of MYmind, Ridderinkhof et al. (2018) employed the CAMM to assess mindfulness in children with autism. In the current sample, internal consistency for the CAMM total score was good (Cronbach’s *α* = .87).

#### Youth Autism Symptoms

Autism symptomatology was assessed using the parent report version of the *Social Responsiveness Scale, Second Edition* (*SRS-2*; Constantino and Gruber 2012). The 65 items were rated on a 4-point response scale (1 = “Not True” to 4 = “Almost Always True”), providing *T*-scores for overall ASD symptom severity, as well as for five subscales: Social Awareness, Social Cognition, Social Communication, Social Motivation, and Restrictive and Repetitive Behaviors. The SRS-2 has been used to measure autism symptoms in past MBT trials and has good psychometric properties in these situations (e.g., de Bruin et al. 2015; Ridderinkhof et al. 2018). In the current study, internal consistency was excellent for overall severity (Cronbach’s *α* = .93) and was acceptable to good for all subscales (Cronbach’s *α* = 75–.87), except for Social Awareness (Cronbach’s *α* = .33).

#### Parent Mental Health

The 21-item *Depression, Anxiety, and Stress Scale* (*DASS-21*; Lovibond 1995) was used to measure parent psychopathology and stress. Parents were asked to rate how they had been feeling over the previous week using a 4-point response scale (0 = “Did not apply to me at all/Never” and 3 = “Applied to me very much/Almost always”). The DASS-21 is comprised of three subscales: Depression, Anxiety, and Stress. For the current sample, internal consistency ranged from good to excellent for subscales (Cronbach’s *α* = .84–.94) and was excellent for the overall total score (Cronbach’s *α* = .96).

#### Parent Mindfulness

The Five Facets of Mindfulness Questionnaire-Short Form (FFMQ-SF; Bohlmeijer et al. 2011) is a 24-item self-report questionnaire to tap dimensions of  mindfulness. Parents were asked to indicate the frequency of everyday experiences in the last month using a 5-point response scale (1 = “Never/Very Rarely True” to 5 = “Very Often/Always True”). The FFMQ-SF consists of five subscales: Observing, Describing, Acting with Awareness, Non-Judging of Inner Experience (Non-Judge), and Non-Reactivity to Inner Experience (Non-React). Subscale internal consistencies in this sample ranged from fair to good (Cronbach’s *α* = .72–.82) and were acceptable (Cronbach’s *α* = .63) for the overall score.

The Interpersonal Mindfulness in Parenting Scale (IEM-P; Duncan 2007) was used to assess parents’ awareness, present-centered attention, and non-judgment specifically during interactions with their child (e.g., “I often react too quickly to what my child says or does”). Parents provided responses on a 5-point scale (1 = “Never True” to 5 = “Always True”). A total score was calculated by computing the average rating across the ten items. Internal consistency for the total score in the current sample was acceptable (Cronbach’s *α* = .60).

#### Social Validity

A short 9-item survey was developed by the authors to evaluate youth and parent perceived improvement after the MYmind program. The survey was completed online 3–5 months following the booster session. Parents and youth were asked to rate their own and their family member’s progress in managing stress, managing negative emotions, experiencing quality of life, and whether the relationship with their family member had improved. Participants were asked to respond on a 3-point scale (1 = “Yes,” 2 = “Sometimes,” or 3 = “No”). The survey also asked an open-ended question about how helpful the program was, and whether participants experienced any barriers while participating in the MYmind program.

### Data Analyses

Multilevel modeling was used to evaluate change over time for each outcome measure. Assessment time points were included as predictors. The 1-week pre-program score was consistently used as the control time point and was compared with the 10-week pre-program score to assess change during the baseline period; the 1-week post-program score to assess change during the program period; and the 10-week post-program score to assess change at follow-up. All measurement time points were listed as fixed, nested within participants (either parent or youth depending on the measure), with a random intercept. Parameter estimates are indicative of change from 1 week prior to beginning the program (i.e., model intercept). Following methods employed by Ridderinkhof et al. (2018), data were standardized in order to interpret parameter estimates as Cohen’s *d* effect sizes, with 0.20, 0.50, and 0.80 being considered small, medium, and large effects, respectively. Given the risk of type II error associated with clinical pilot work (Leon et al. 2011), no corrections for type I error were applied. Participant feedback on the program was analyzed using descriptive statistics.

## Results

Multilevel model results of youth outcomes are presented in Table 2, and parent outcomes in Table 3.

**Table 2:** Parameter estimates of outcome variables for adolescents

	Effects over time			
	Intercept^a^	10 weeks pre	1 week post	10 weeks post
BASC-SRP				
School Problems	0.12 (0.21)	− 0.21 (0.14)	− 0.17 (0.14)	− 0.07 (0.14)
Internalizing	0.02 (0.21)	− 0.00 (0.09)	− 0.04 (0.09)	− 0.01 (0.09)
Inattention/Hyperactivity	0.06 (0.21)	− 0.11 (0.09)	− 0.15 (0.09)^+^	− 0.11 (0.09)
Emotion Symptoms Index	0.03 (0.21)	0.01 (0.10)	− 0.09 (0.10)	− 0.05 (0.10)
Personal Adjustment	− 0.00 (0.21)	− 0.05 (0.12)	0.22 (0.12)^+^	− 0.00 (0.12)
BASC-PRS^b^				
Externalizing Problems	0.13 (0.20)	0.06 (0.15)	− 0.28 (0.15)^+^	− 0.25 (0.15)^+^
Internalizing Problems	0.07 (0.20)	0.08 (0.13)	− 0.13 (0.13)	− 0.14 (0.13)
Behavioral Symptoms Index	0.10 (0.20)	0.14 (0.14)	− 0.29 (0.14)*	− 0.24 (0.14)
Adaptive Skills	− 0.19 (0.20)	− 0.02 (0.11)	0.24 (0.11)*	0.36 (0.11)**
RRS				
Reflection	− 0.16 (0.21)	0.07 (0.11)	0.31 (0.11)**	0.26 (0.11)*
Brooding	− 0.00 (0.20)	0.08 (0.14)	− 0.11 (0.14)	0.14 (0.14)
Depression	− 0.11 (0.20)	0.02 (0.12)	0.07 (0.12)	0.33 (0.12)**
Total	− 0.11 (0.20)	0.06 (0.11)	0.10 (0.11)	0.32 (0.11)**
ERQ-CA				
Cognitive Reappraisal	− 0.15 (0.21)	0.05 (0.18)	0.45 (0.18)*	0.24 (0.18)
Expressive Suppression	− 0.04 (0.20)	0.24 (0.15)	0.04 (0.15)	− 0.05 (0.15)
ERC^b^				
Lability/Negativity	0.10 (0.21)	0.15 (0.17)	− 0.29 (0.17)^+^	− 0.19 (0.17)
Emotion Regulation	− 0.12 (0.21)	0.04 (0.13)	0.24 (0.13)^+^	0.29 (0.14)*
CAMM				
Total	− 0.09 (0.21)	0.01 (0.13)	0.04 (0.13)	0.08 (0.13)
SRS-2^b^				
Social Awareness	− 0.03 (0.20)	0.13 (0.18)	− 0.12 (0.18)	0.07 (0.18)
Social Cognition	− 0.02 (0.21)	0.24 (0.17)	− 0.19 (0.17)	− 0.05 (0.17)
Social Communication	0.05 (0.21)	0.02 (0.13)	− 0.24 (0.13)^+^	− 0.22 (0.13)^+^
Social Motivation	0.18 (0.20)	0.12 (0.15)	− 0.35 (0.15)*	− 0.59 (0.15)**
RRBs	0.12 (0.19)	0.18 (0.14)	− 0.23 (0.15)	− 0.30 (0.15)*
Total score	0.08 (0.20)	0.14 (0.15)	− 0.32 (0.15)*	− 0.30 (0.15)^+^

*BASC-PRS*, Behavior Assessment System for Children, Second Edition – Parent Rating Scales; *BASC-SRP*, Behavior Assessment System for Children, Second Edition – Self-Report of Personality; *CAMM*, Child and Adolescent Mindfulness Measure; *ERQ-CA*, Emotion Regulation Questionnaire for Children and Adolescents; *RRB*, Restrictive Repetitive Behaviors subscale; *RRS*, Rumination Response Scale; *SRS-2*, Social Responsiveness Scale, Second Edition

^a^Comparison group = 1 week pre

^b^Parent-reported outcomes

**p < .01; *p < .05; ^+^*p* < .10

**Table 3:** Parameter estimates of outcome variables for parents

	Effects over time			
	Intercept (*SE*)	10 weeks pre (*SE*)	1 week post (*SE*)	10 weeks post (*SE*)
FFMQ				
Observe	− 0.19 (0.20)	0.23 (0.12)^+^	0.36 (0.12)**	0.34 (0.12)**
Describe	0.04 (0.20)	− 0.22 (0.12)^+^	0.12 (0.12)	0.21 (0.12)^+^
Acting with Awareness	− 0.05 (0.21)	0.01 (0.19)	0.27 (0.19)	0.19 (0.19)
Non-Judging	− 0.07 (0.21)	− 0.06 (0.18)	0.20 (0.18)	0.28 (0.18)
Non-Reacting	− 0.12 (0.20)	0.08 (0.18)	0.18 (0.18)	0.27 (0.18)
Total	− 0.11 (0.21)	− 0.01 (0.16)	0.36 (0.16)*	0.40 (0.16)*
DASS				
Depression	− 0.12 (0.20)	0.26 (0.15)^+^	− 0.10 (0.15)	0.14 (0.16)
Anxiety	0.04 (0.23)	0.22 (0.18)	− 0.10 (0.18)	− 0.08 (0.19)
Stress	− 0.03 (0.20)	0.31 (0.18)^+^	− 0.18 (0.18)	− 0.06 (0.19)
Total	− 0.06 (0.21)	0.31 (0.17)^+^	− 0.14 (0.16)	0.02 (0.17)
IEM-P				
Total	− 0.05 (0.20)	− 0.09 (0.15)	0.04 (0.15)	0.31 (0.15)*

*DASS*, Depression, Anxiety and Stress Scale; *FFMQ*, Five Facet Mindfulness Questionnaire; *IEM-P*, Interpersonal Mindfulness in Parenting Scale

**p < .01; *p < .05; ^+^*p* < .10

### Youth Mental Health

During the baseline period, no significant changes were reported by youth or parents on measures of youth mental health. After completing the program, parents reported a small significant decrease on the BASC-2 Behavioral Symptoms Index; however, change was no longer significant at follow-up (i.e., gains were not maintained). Parents also reported a small significant increase in BASC-2 Adaptive Skills post-program and at follow-up. There were no significant changes in parent-reported Internalizing Problems at post-program or follow-up, and only trends towards significance with regard to externalizing symptoms at post-program and follow-up. Youth did not report significant changes on any BASC-2 composites, at any time point.

### Youth Emotion Regulation

As expected, there were no significant changes during the baseline period on RRS, ERQ, or ERC subscales. Following the program, youth reported significant changes on RRS Reflection, with a small effect size, which was maintained at follow-up. While youth did not report significant changes on RRS Brooding, Depression, or Total scores at 1-week post-program, there were significant improvements for RRS Depression and RRS Total at follow-up. Significant improvements on the ERQ’s Cognitive Reappraisal subscale was found at post-program, though these were not maintained at follow-up, and no significant changes were observed on the Expressive Suppression subscale.

According to parents, there was a trend towards improvement in youth ERC Emotion Regulation skills at post-program, which emerged as significant at follow-up. At the same time, reductions in youth emotional lability (ERC Lability/Negativity) only reached the level of a trend at post-program, and this failed to be maintained at follow-up.

### Youth Mindfulness

There were no significant changes in youth-reported mindfulness (CAMM) in any of the time periods.

### Autism Symptoms

There were no significant changes on any SRS-2 subscales during the baseline period. Immediately following the program, parents reported improvements in Overall autism symptoms and in Social Motivation, which were maintained at follow-up, with small to medium effects. There was also a trend indicating improvements in Social Communication at both of these time points, and a significant improvement in Restrictive and Repetitive Behaviors at follow-up. There were no significant changes in Social Awareness or Social Cognition.

### Parent Mental Health

Contrary to expectations, parents did not report significant changes in the program or follow-up periods for any DASS subscale. Further, while there were no significant changes on DASS subscales during the baseline period, there were trends indicating improvements in this phase for Depression, Stress, and Overall symptoms.

### Parent Mindfulness

As expected, there were no significant changes in the FFMQ subscales (though there were some trends for two subscales), or the IEM-P total score, during the baseline period. Parents reported significant improvements for FFMQ Observe facet and Total scores at 1-week post-program, which were maintained at follow-up. There were no significant changes reported at post-program or follow-up for the other FFMQ subscales. While there was no change in IEM-P Overall score immediately following the program, significant improvement was noted at follow-up.

### Social Validity

Of the 19 parents who completed the social validity questionnaire, none indicated experiencing any barriers to participating in MYmind, and 15 provided open-ended feedback of experiences, such as “It has given [youth] a valuable tool to help her cope with anxieties. She continues to have challenges, however, and we are continuing to practice and use the strategies we learned in the program.” Broader benefits of parent social support were suggested: “…the parent discussions were extremely helpful and supportive. There is a great need for parents of these students to get together in support groups.” Participants also mentioned that discontinuation of the group made maintaining gains more difficult. One parent stated: “…The hardest part is that the program didn’t put in a routine that we can easily continue,” while another parent noted: “It [mindfulness] was helping me de-stress while the sessions were on. It is more difficult to maintain without the group.”

Of the 16 youth who completed the social validity questionnaire, 9 provided open-ended feedback about how participating in MYmind impacted them. Examples of positive feedback included “It has helped me to learn to be in the moment and know that it is okay to walk out of a situation, to breathe, and then go back into that situation whatever it may be,” and, “The MYmind program has helped me to be a more calm and focused person.” Many participants reported the benefits of socialization and connectedness to the community. One youth noted: “I was able to meet other autistic people for the first time in my life, which finally made me feel like I wasn’t alone - there are others like me out there. That in and of itself was an empowering thing to have.” Examples of negative feedback to the question about anything being helpful included “Not really. That’s just how it is. But the program has been interesting nonetheless,” and, “Most of the factors that drove my self-esteem up were external to the program.”

**Fig. 2:**
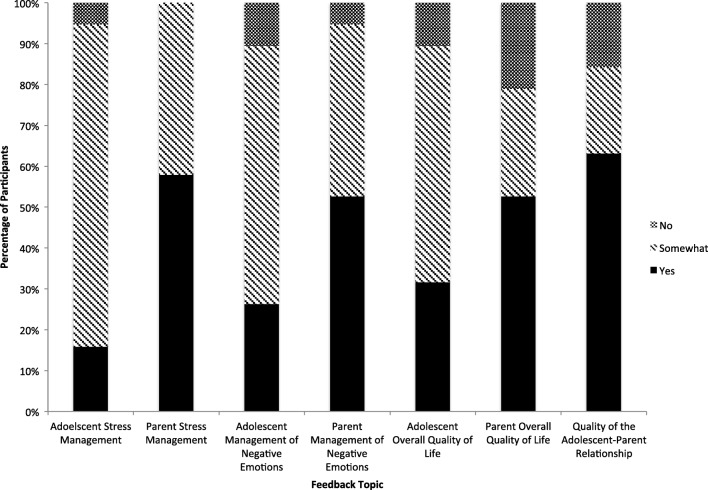
Parent report on management of stress, negative emotion, and quality of life

**Fig. 3:**
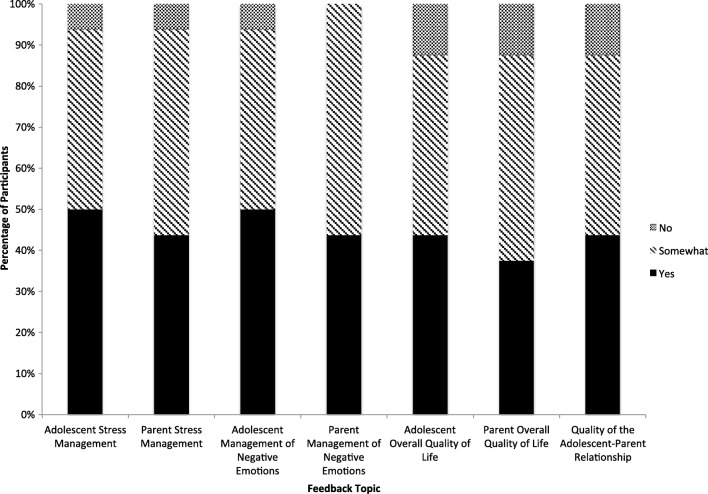
Youth report on management of stress, negative emotion, and quality of life

As shown in Figs. 2 and 3, according to the survey, most participants felt that there was at least some improvement in their own and their family member’s management of stress and negative emotions. About 89% of youth and 79% of parents believed that quality of life and relationship quality within their dyad were also at least somewhat improved. The lowest noted improvement rating was for parent self-report on quality of life; 21% of parents believed they had gained no improvement in their quality of life.

## Discussion

This is the third study to examine parent and youth changes following participation in MYmind, a concurrent MBT program designed for individuals with autism and their parents. It builds on de Bruin et al. (2015) and Ridderinkhof et al. (2018), by including a baseline comparison, measures of mental health and emotion regulation, and being completed by an independent research group.

The current results are partially in line with these previous two studies. In all trials, parents showed significant changes in mindfulness over the course of the program. According to parent self-report, the Overall FFMQ score and Observe facet increased significantly in the program phase. In addition, the overall mindful parenting measure improved from pre-program to follow-up, though not at post-program. Similarly, de Bruin et al. (2015) found improvements in the parents’ ability to be less reactive, observe and describe their emotions, and listen to their children with full attention and without judgment at follow-up, though the effects related to non-judgment did not last into follow-up. Ridderinkhof et al. (2018) noted improvement in mindful parenting and self-compassion at post-test, 2-month, and 1-year follow-ups. Although overall parent mindfulness seemed to change in all three studies, specific facets vary. For example, de Bruin et al. (2015) found greater changes in many of the facets of mindfulness as measured on the FFMQ relative to the current study. While we maintained fidelity to the program manual, the MYmind protocol allows for variations in the distribution of teaching and participant home practice, as well as participant engagement in sessions, which may have resulted in differences between sessions, in turn, leading to varied response patterns. For example, participants are often given the choice of which meditations to practice and incorporate into their lives, and by choosing one meditation over another or generally how much they did or did not practice any of the parts of the program, they may practice more or less of certain facets over others. It is also possible that we would have seen more significant effects with a larger sample size.

Youth reported no changes in mindfulness in this trial, or in past MYmind trials (de Bruin et al. 2015; Ridderinkhof et al. 2018). Considering MYmind is meant to directly target mindfulness, this lack of improvement can either point to a problem with assessment and understanding of mindfulness in this population or the program’s inability to impact this process. There is a need to effectively define and measure mindfulness within the context of autism, in individuals who may struggle with accessing or reporting their internal experiences, as well as have issues with using tools designed for the general population (Bishop 2002; Cachia et al. 2015; Hwang and Kearney 2014; Maisel et al. 2016). It is also possible that MYmind’s effects on emotion regulation and mental health occur via mechanisms other than mindfulness, such as elements of the group experience, mindful parenting, or increased relaxation or decreased stress (Ridderinkhof et al. 2018).

Parent-reported improvement in autism symptoms was also in line with the findings of de Bruin et al. (2015) and Ridderinkhof et al. (2018). Parents noted improved overall autism symptoms and social motivation at post-program and follow-up (and trends towards significance in social communication), along with significant improvements in Restrictive and Repetitive Behaviors at follow-up. de Bruin et al. (2015) pointed to improvements in theory of mind for this population as a potential reason for these improvements, citing that in non-ASD samples, mindfulness results in increased empathy and sensitivity to the emotional state of others. It may indeed be that heightened awareness of themselves, others, and the broader social context results in improved social responsiveness and reciprocity in youth, though the directionality of these changes still needs to be tested in other longitudinal designs.

We did find improvements in some aspects of emotion regulation during the program and at follow-up, though some of the immediate post-program changes failed to be maintained. The variables that showed the most change appeared to reflect a positive orientation to have control over one’s emotionally linked thoughts and an ability to change them when needed. In contrast, youth did not report changes in expressive suppression, a scale that reflects experiencing negative emotions by holding them in. In mindfulness, individuals are taught to be aware of their thoughts and feelings, and not to avoid or suppress them. Youth also reported improvements in aspects of rumination, including their tendency to contemplate and attempt to deal with problems or difficulties (though this was not maintained at follow-up), cognitions related to sadness and hopelessness, and self-reflection and repetitive focus on negative emotions, though some changes only emerged at follow-up. Parents reported improvements on the emotion regulation subscale of the ERC, which reflects a positive effect on their children’s emotional expression, empathy, and emotional self-awareness. In contrast, parents did not report significant changes with regard to Lability/Negativity, which reflects an inability to regulate anger, engage in flexible thinking, and experience fewer dramatic changes in mood. Taken together, this suggests that youth may be more able to demonstrate positive emotions, though they may still struggle with response moderation and the appropriate expression of negative emotions. This finding is in line with previous research on MBT outside of the autism population, which suggests that children demonstrate more awareness of their positive emotions and of the way they express them (Dumas 2005; Hwang et al. 2015; Khaddouma et al. 2015). It is possible that MYmind’s focus on the present moment without judgment fosters self-awareness and acceptance of actions, emotions, and thoughts, which is related to the ability to limit perseveration about past and future events.

In contrast, we found few effects on broader measures of youth psychopathology. In fact, there were no changes in parent reports of youth internalizing symptoms, and only trends with regard to externalizing symptoms, or youth self-report of internalizing issues, inattention/hyperactivity, or overall emotional disturbance. This finding differs from earlier reviews that suggest that MBT may reduce internalizing problems in adolescents and adults with autism (Cachia et al. 2016). An examination of the *T*-scores may reflect why there were no observed changes in these symptoms. At baseline, youth-reported scores across all composites were in the average range, suggesting that they perhaps were not sufficiently self-aware and/or did not identify with problems in these components to begin with. Similarly, for parent report, scores on externalizing symptoms were also in the average range, while scores on internalizing symptoms were deemed “At-Risk” but not at a “Clinically Significant” level. Youth were not required to have clinically significant emotional or behavioral problems to participate in this program, and thus a lack of change may reflect this sample bias. Future research should examine the application of MBT exclusively in youth with autism who have clinically significant emotional and behavioral problems. According to parents, youth improved and maintained their Adaptive Skills over the course of the program, which measures adaptability, skills associated with functioning in daily life, improved coping strategies, and feelings of control (e.g., activation of daily living, adaptability, functional communication, leadership, social skills, and study skills) rather than psychopathology per se. It is possible that within the context of this program, in youth with low levels of psychopathology, that the application of mindfulness either positively impacts their openness to engage in more socially appropriate behaviors, be more effective in response to changes, or that parents are more aware of indeed what their children can do.

Parents reported trends towards improvement in stress, depression, and overall mental health in the baseline phase only. Prior studies have suggested that mindfulness for parents of children with autism can improve their stress levels (de Bruin et al. 2015; Ridderinkhof et al. 2018). However, these past studies did not include a repeated measures control condition, and our lack of significance may reflect our more stringent research design. Reported improvements during the baseline phase suggest that changes in mental health could represent non-program variables, such as time, re-assessment, or the impact of hope/expectation (that comes with the anticipation of receiving support/potentially incurring benefit) on well-being and connecting with other parents (Gallagher and Lopez 2009). It is critical to next include randomized controlled trials to further assess whether some degree of reported mental health improvement is indeed an artifact.

This program was found to have a high level of feasibility. Attendance rates, fidelity, and participant feedback were generally very positive. Participants attended approximately 90% of sessions in the trial, and attrition was relatively low. This reflects participant commitment to, and positive belief about, the benefits of the program. Most participants felt that they were better able to manage stress and negative emotions at least to some degree. Overall, youth and parents perceived quality of life gains, and most also believed that relationships within their dyad had improved following the program.

It is acknowledged that the MYmind program was relatively short, at 9 weeks in length, and some parents felt that it either ended too quickly or did not sufficiently help them incorporate mindfulness practice firmly in their daily lives. While positive feedback lends support for MYmind, it is important to acknowledge that participant reported change following the program may not extend beyond what we would anticipate from chance, and are subject to positive biases, as those who did not complete the post-program satisfaction survey may be the least satisfied with it. These reported changes might also be due to factors common to all psychosocial interventions, rather than to the specific activities of MBT.

## Limitations and Future Research

Study results seem to support MYmind as a potential mindfulness training program for parents and youth with autism; however, there are some limitations to this study. First, as a result of the study time frame, different facilitators and co-facilitators delivered the program for each group. Even though each facilitator was required to have completed MYmind training, the inclusion of several facilitators with a range of experience in mindfulness could impact the delivery; as such, assessing and addressing treatment fidelity in situ would be important to accurate implementation. Second, although a within-subject repeated measures design offers some methodological strengths, it is critical to incorporate more stringent control conditions and randomization procedures like those in an RCT. Third, outcome measures used here may be highly influenced by the respondent bias. The measures rely on youth and parent report and did not include any direct measures, which is a common failing in MBT research (Burke 2010). Behavioral observation would be beneficial to provide direct measurement of observable behavioral changes, as opposed to perceived changes in behavior. Additionally, all measures were presented multiple times and so test-retest effects may impact the results. Fourth, given the discrepancies between participant noted changes and changes in measurement, this may point to a lack of a degree of sensitivity of the measures. We also did not track the degree of home practice in youth or parents, which limits our ability to determine whether home activities have a role to play in change. Fifth, the sample size was relatively small and included a wide range of ages, which were chosen to reflect the original de Bruin et al. (2015) study. These factors may have impacted statistical analysis and introduced some confusion of results as participants of different ages could be impacted differently. Finally, the statistical methodologies used here result in an increased probability of type I error. These limitations provide additional opportunities for future research.
